# Framework for the treatment and reporting of missing data in observational studies: The Treatment And Reporting of Missing data in Observational Studies framework

**DOI:** 10.1016/j.jclinepi.2021.01.008

**Published:** 2021-06

**Authors:** Katherine J. Lee, Kate M. Tilling, Rosie P. Cornish, Roderick J.A. Little, Melanie L. Bell, Els Goetghebeur, Joseph W. Hogan, James R. Carpenter, the STRATOS initiative

**Affiliations:** a Clinical Epidemiology and Biostatistics Unit, Murdoch Children's Research Institute, Melbourne, Australia; b Department of Paediatrics, University of Melbourne, Melbourne, Australia; c MRC Integrative Epidemiology Unit, University of Bristol, Bristol, UK; d Department of Statistics, University of Michigan, MI, USA; e Department of Epidemiology and Biostatistics, University of Arizona, AZ, USA; f Department of Applied Mathematics, Computer Science and Statistics, Ghent University, Ghent, Belgium; g Department of Biostatistics, Brown University, RI, USA; h MRC Clinical Trials Unit, London School of Hygiene and Tropical Medicine, London, UK

**Keywords:** Missing data, Multiple imputation, Observational studies, Reporting, ALSPAC, STRATOS initiative

## Abstract

Missing data are ubiquitous in medical research. Although there is increasing guidance on how to handle missing data, practice is changing slowly and misapprehensions abound, particularly in observational research. Importantly, the lack of transparency around methodological decisions is threatening the validity and reproducibility of modern research. We present a practical framework for handling and reporting the analysis of incomplete data in observational studies, which we illustrate using a case study from the Avon Longitudinal Study of Parents and Children. The framework consists of three steps: 1) Develop an analysis plan specifying the analysis model and how missing data are going to be addressed. An important consideration is whether a complete records’ analysis is likely to be valid, whether multiple imputation or an alternative approach is likely to offer benefits and whether a sensitivity analysis regarding the missingness mechanism is required; 2) Examine the data, checking the methods outlined in the analysis plan are appropriate, and conduct the preplanned analysis; and 3) Report the results, including a description of the missing data, details on how the missing data were addressed, and the results from all analyses, interpreted in light of the missing data and the clinical relevance. This framework seeks to support researchers in thinking systematically about missing data and transparently reporting the potential effect on the study results, therefore increasing the confidence in and reproducibility of research findings.

## Highlights

• Missing data are ubiquitous in medical research.
• Guidance is available, but missing data are still often not handled appropriately.
• We present a framework for handling and reporting analyses of incomplete data.
• This framework encourages researchers to think systematically about missing data.
• Adoption of this framework will increase the reproducibility of research findings.
• This article provides a much needed framework for handling and reporting the analysis of incomplete data in observational studies.
• The framework puts a strong emphasis on preplanning the statistical analysis and encourages transparency when reporting the results of a study.
• Adoption of this framework will increase the confidence in and reproducibility of research findings.

## 1 Background

Despite recent reviews emphasizing the need to minimize missing data during the design stage [1], missing data remain ubiquitous in medical research. For example, in the Avon Longitudinal Study of Parents and Children (ALSPAC), a transgenerational prospective observational study of 14,500 families in the United Kingdom, only 48.2% of children completed the 12 measures collected during adolescence. Electronic routinely collected data sets, which are increasingly exploited in observational research, are particularly susceptible to missing data because data are collected for clinical reasons, rather than designed research.

Despite increasing guidance on how to handle missing data [2,3], practice is changing slowly and misapprehensions abound. This is particularly pertinent in observational research [4,5], where there is no regulatory framework guiding the analysis, and analyses are often adjusted for confounders which can have missing values. Restricting analysis to records with complete data for the analysis model (termed complete case or complete records analysis) is still the most common approach based on our experience and in the latest systematic reviews we are aware of [6,7], although it is known to result in a loss of power and in many situations will cause bias. Yet, researchers often do not consider the potential impact of missing data on their scientific conclusions [8]. This is despite journals requiring justification for the method used to handle missing data [9] and tools for assessing the quality of studies having domains referring to how missing data were addressed.

Multiple imputation (MI) is a practical, flexible approach for handling missing data [10] that is becoming increasingly popular [11,12]. Under this approach, missing values are imputed from the predictive distribution of the missing given observed data multiple times. Next, the analysis model is fitted to each “complete” data set and the results combined using Rubin's rules [10]. A key benefit of MI is that it can readily incorporate *auxiliary* variables (variables predictive of missing values but not in the substantive model) into the imputation step; this can often reduce bias and improve efficiency. MI is available in all leading statistical software packages. However, this ease of use may result in MI being applied without proper consideration of its appropriateness and fundamental mistakes being made [13,14]. Moreover, MI may not always provide a preferable method of handling missing data [15] as we will illustrate in this manuscript.

In this article, we propose our Treatment and Reporting of Missing data in Observational Studies (TARMOS) framework, a practical framework for researchers faced with analyzing incomplete observational data. We focus on MI because of its flexibility and prominence in the literature, although—as we discuss later—similar principles apply to any approach for handling missing data.

First, we describe a case study from the ALSPAC. We then present our framework, illustrating each step in turn. Although we focus on a simple exposure-outcome relationship, the principles underpinning our framework apply quite generally.

## 2 Case study: The avon longitudinal study of parents and children

The ALSPAC recruited pregnant women living in and around Bristol, England, in the early 1990s. The study has been described previously [16,17]. Briefly, 14,541 women were initially recruited, resulting in 14,062 live births and 13,988 children alive at 1 year; additional children were enrolled subsequently. ALSPAC has a fully searchable data dictionary and variable search tool (http://www.bristol.ac.uk/alspac/researchers/our-data/). Ethical approval was obtained from the ALSPAC Ethics and Law Committee and the Local Research Ethics Committees.

The ALSPAC suffers from attrition and sporadic missingness. Attrition was highest in infancy and late adolescence, and previous analyses have shown that those who continue to participate are more likely to be female, white, and live in high-income households [16].

Our case study assesses whether there is an association between smoking at 14 years and educational attainment at 16 years. This is a modified version of the research question published previously [18]. The analysis used data from 14,684 adolescents—the full cohort less than those who died or withdrew consent before 14 years, but there are missing data in all variables required for analysis (except sex). Stata code for the case study is given in the Supplementary Material.

### 2.1 Outcome

Educational attainment score at 16 years obtained via linkage to the National Pupil Database (https://www.gov.uk/government/collections/national-pupil-database). The score is the percentage of the maximum observed in the data (540 points).

### 2.2 Exposure

Participants were asked about smoking via a computerized questionnaire during a clinic assessment (mean age 13.8 years) and a postal questionnaire (mean age 14.1 years). Both included questions about past and current smoking which were used to classify individuals as current or nonsmokers.

### 2.3 Additional variables

Data were collected on several potential confounding variables capturing education and related social factors at recruitment and auxiliary variables, largely measured at other waves of data collection (See Table 1 and Supplementary Table 1).

**Table 1:** Summary of variables for analysis

Variable type	Definition	Relevant variable(s) in the ALSPAC case study
Outcome	Outcome of interest in the analysis model.	Educational attainment score at 16 years
Exposure	Main exposure of interest in the analysis model.	Smoking status at 14 years
Confounders	Variables required for adjustment in the analysis model.	Child sex Parity Maternal smoking status Paternal smoking status Maternal educational level Paternal educational level Behavioral difficulties score at 81 months Attainment score at 11 years
Auxiliary	Variables that are not in the analysis model but can be used to recover some of the missing data in the incomplete variables	Smoking age 10 years Smoking age 13 years Frequency of smoking at 15 years IQ age 8 years Behavior score at 57 months Duration of breastfeeding Number of rooms in home (excluding bathrooms) during pregnancy Family occupational social class (higher of maternal and paternal) Car ownership Housing tenure

## 3 The framework

**Fig. 1:**
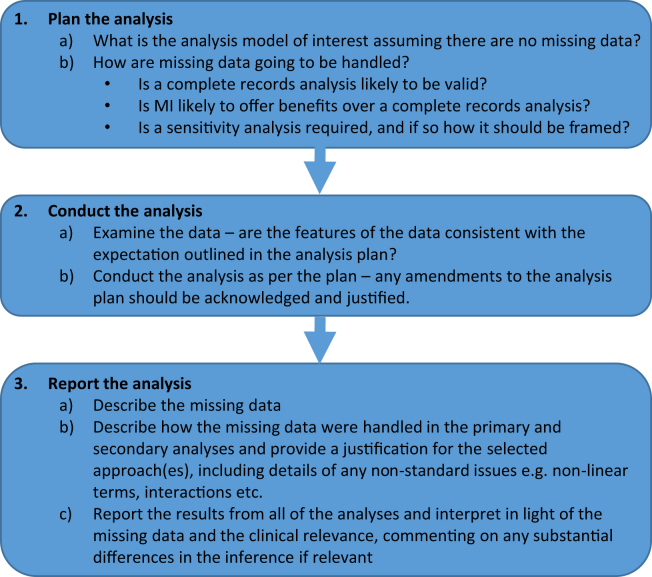
The framework.

Figure 1 outlines our framework. Below, we describe the steps of this framework.

### 3.1 Step 1: Plan the analysis

When designing a research study, it is important to prespecify an analysis plan stating the primary and any secondary analyses (prospectively for prospectively collected data). In much observational research, (e.g., our case study), the data will have already been collected. In this context, there may be knowledge about the data, including levels of missingness and potential missingness mechanisms, which can be used to develop the analysis plan. If there is little or no prior information about the missing data, for example, if using electronic health records, a general plan should still be outlined, but the plan may be contingent on the missing data and the relationships between the complete and incomplete variables.

#### 3.1.1 Step 1a. Identify the substantive research question(s) and plan the statistical analysis

The first step is to identify the substantive research question(s), that is, the exposure(s), outcome(s), causal structure (if relevant), confounders, and corresponding analysis model(s). This should (generally) be performed without consideration of the missing data. In ALSPAC, the target quantity is the mean difference in educational attainment in smokers versus nonsmokers, and our analysis model is a linear regression of educational attainment at 16 years on smoking at 14 years adjusted for confounders outlined in Supplementary Table 1. For simplicity, we assume this is a valid analysis model for our question.

#### 3.1.2 Step 1b. Specify how the missing data will be addressed

Decisions concerning missing values should be informed by their most plausible contextual cause. For a single incomplete variable, this is often linked to Rubin's typology [19]:

• Missing completely at random (MCAR)—missingness does not depend on anything related to the substantive research question, e.g., missingness dependent on wave of data collection in a cross-sectional analysis;
• Missing at random (MAR)—missingness may depend on its value, but this dependence is broken within strata of (i.e., conditional on) fully observed variables, for example, missingness on smoking dependent on smoking status, but not after stratifying by social class (which has no missing data); and
• Missing not at random (MNAR)—even within strata of observed variables, missingness still depends on the value itself, for example, within social strata, missing smoking data depends on smoking status.

**Fig. 2:**
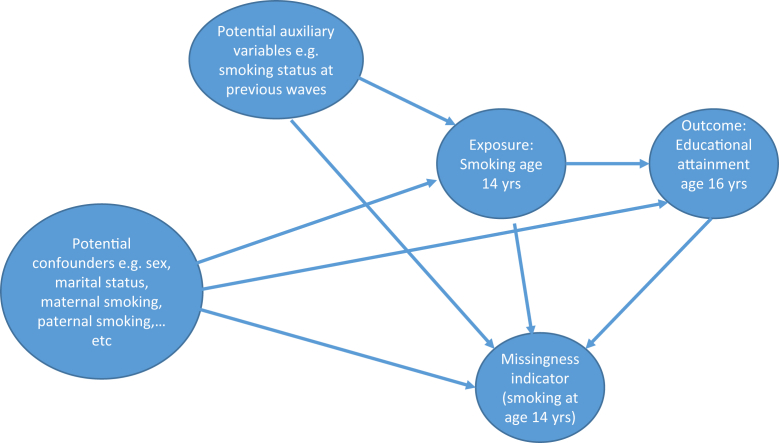
Causal diagram for the Avon Longitudinal Study of Parents and Children (ALSPAC) case study. Note, this figure illustrates the fact that we expect missingness to depend on the outcome of interest, educational attainment, as well as smoking itself, and that we expect there will be potential auxiliary variables that are both associated with missingness and with the incomplete exposure variable (smoking age 14 years).

Although this classification is useful when there is a single incomplete variable, it is not straightforward when there are multiple incomplete variables. A more natural way to understand the assumptions regarding missing data for a given research question where there are multiple incomplete variables is to use causal diagrams [15,20,21]. See Figure 2 for a causal diagram for the ALSPAC case study.

**Fig. 3:**
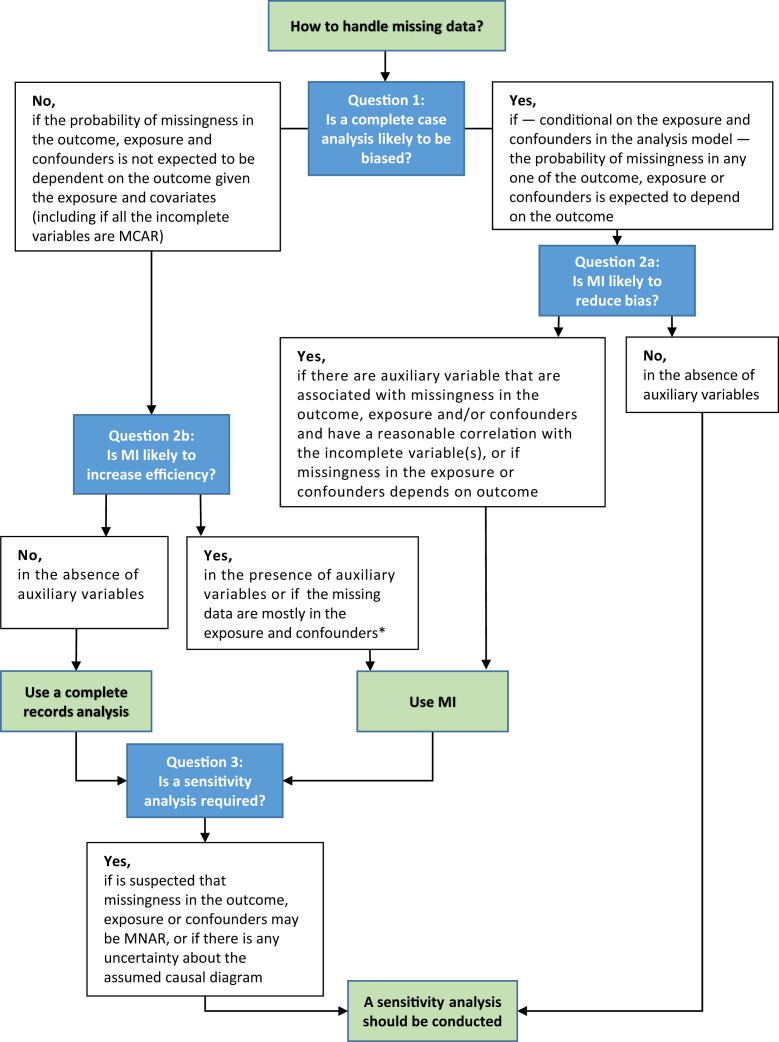
Flowchart for selecting an appropriate method to handle the missing data. ∗ The exception is if there is missingness in the exposure or covariates that is unrealted to the outcome but is missing not at random; in this context although inference from a complete records analysis would be unbiased, inference following multiple imputataion would be biased.

Figure 3 provides an overview of the decision-making process regarding missing data when estimating an exposure-outcome association as in our case study. We propose three key questions to guide the process:

**Q1: Is a complete records analysis likely to give valid inference for the exposure effect? This will depend on:**

• ***How much information is expected to be lost because of missing values:*** This will depend on which variables are incomplete, the proportion of missing data and the information retained by auxiliary variables. If there is unlikely to be much missing information in the exposure, outcome, and key confounders (e.g., if <5% of records are expected to have missing values), it will not make much difference how missing data are handled, irrespective of auxiliary variables, and a complete records analysis might be acceptable [22]. If, however, there is more missing information, for example, more incomplete records, then MI may be more efficient. This may not be true if there is only missingness in the outcome, and there are no auxiliary variables, as noted below.
• ***What are the likely mechanisms behind missing data:*** There are a range of situations under which a complete records analysis is likely to be unbiased for linear and logistic regression models; these have been outlined in the literature [15,20,23,24]. Importantly, a complete records analysis will be unbiased for estimating a correctly specified exposure-outcome relationship if the reasons for missingness in any variable in the analysis model is not related to the outcome (given the other variables in the analysis model), although it may still be inefficient. This is true even if the missingness in the exposure or covariates is MNAR [15].

The analysis plan may specify that the strategy for dealing with missing data will depend on the extent of, and reasons for, missing data. For example, the plan could be that if <5% of cases have missing data and there is little evidence that the observed variables are associated with any missingness, then a complete records analysis will be used. If, however, ≥5% of cases have missing data and there is evidence that data are not MCAR then MI will be used.

Note, if a complete records analysis is not to be the primary analysis, it can still be useful to conduct such an analysis as a sensitivity analysis that makes a different assumption about the missingness.

In ALSPAC, dropout is associated with many of the covariates in the analysis model (i.e., is not MCAR), and in particular educational attainment (the outcome) [16]. Given this, complete records analysis is likely to be biased, and hence would be inappropriate.

**Q2: Is MI (or an inferentially equivalent approach) likely to give a) important bias reduction and/or b) increased precision over a complete records analysis?** This will depend on:

• ***The extent of missing information:*** The more missing information, the greater the potential gains from MI. However, this will be contingent on which variables are incomplete, the appropriateness of the imputation model, and the presence and strength of relationships with auxiliary variables.
• ***Whether there are auxiliary variables that may provide information about the missing values:*** If there are auxiliary variables that are correlated with the incomplete variable(s), including these variables in the imputation model will reduce bias and improve precision over the complete records analysis. In many analyses, there will be a large list of possible auxiliary variables. Typically, it will be best to identify a small number to include, focusing on variables that are strongest, nearly independent predictors of missing values. Variables which predict missingness in one or more variable but are unrelated to the missing values are of less importance [25]. In selecting auxiliary variables, it is important to consider their completeness; their inclusion is only beneficial if they are observed when the variables of interest are missing.
• ***Which variables are likely to contain missing data:*** If most individuals have complete outcome and exposure but incomplete confounders then MI can increase the information about the exposure effect. In contrast, there is less to gain if the missingness is in the exposure and/or outcome [26], unless there are strong auxiliary variables. In the absence of auxiliary variables, MI provides no additional information if only the outcome is incomplete, irrespective of whether exposure/confounders are incomplete. Of note, in the special case where there is an incomplete exposure or confounder that is MNAR but where missingness is unrelated to the outcome, MI would lead to bias despite a complete records analysis being unbiased [15].

As with all statistical models, an improvement in bias and/or precision with MI is contingent on having an appropriately specified imputation model. In particular, the imputation model needs to be compatible with the substantive model—that is, includes the same variables in the same form, including any nonlinear terms and interactions [27]. See [28] for a formal description of compatibility.

In ALSPAC, 51% have missing data on smoking status at 14 years, and we expect missingness to be associated with the outcome. There are a number of strong auxiliary variables, such as smoking status at previous and later waves, which are observed when the exposure of interest is missing in some observations. Given this, MI has the potential to reduce bias and improve precision over a complete records analysis.

**Q3: Is a sensitivity analysis required?** Given that any analysis makes specific (and untestable) assumptions about the missingness mechanism, it is important to explore the robustness of the scientific conclusions to the assumptions [29]. For example, we may wish to carry out an analysis allowing for the fact that data may be MNAR. This could be carried out using MI or using an alternative approach such as assuming that those with missing smoking data are all smokers as we illustrate later. Another form of sensitivity analysis considers the specification of the imputation models, which relies on numerous subjective decisions. This can be important but, for brevity, we restrict our focus to sensitivity analysis regarding the missingness mechanism.

In ALSPAC, we hypothesized that missingness in smoking at 14 would be associated with smoking itself, conditional on the covariates in the analysis model (i.e., MNAR), hence we specify that we will conduct a sensitivity analysis.

#### 3.1.3 Step 1c. Provide details on how the MI will be conducted (if required)

If the analysis plan states that MI (or an alternative MAR method) will be used to handle the missing data, it is important to detail exactly how the analysis will be conducted (including justification) in the analysis plan. For MI, this should include the method of imputation, the variables to be included in the imputation model, the form of variables to be imputed, the nature of the relationships between the variables including any nonlinear relationships and interactions, the method of imputation (e.g., multivariate normal imputation [30], fully conditional specification [31,32], predictive mean matching etc.), the number of imputations, and the software to be used.

See the supplementary material for example text for our case study.

#### 3.1.4 Step 1d. Provide details on how the sensitivity analyses will be conducted (if required)

Sensitivity analyses can rapidly get very complex, hence it is common to focus on one or two contextually important variables, e.g., the outcome and/or exposure of interest (if a nontrivial proportion of missing values) or the confounder(s) with the largest proportion of missing data.

In a sensitivity analysis, we need to change the dependency of the missing values on the other variables, typically the outcome, exposure, or the incomplete variable itself. This can sometimes be performed quite simply. For example, in ALSPAC, individuals with observed data on smoking at 14 years were less likely to report ever having smoked at 10 and 13 years compared with those with missing data (0.8% vs. 3.2% at 10 years and 10.0% vs. 29.3% at 13 years). Thus, as an initial, relatively crude, sensitivity analysis, we could explore what happens when smoking is always imputed as “1” [33,34]. If this has limited effect, a more subtle approach is not required. However, when this extreme assumption has a strong effect, we may need to explore more plausible mechanisms.

A simple way to allow different relationships in the complete and incomplete records is using a pattern-mixture approach [35,36], where, for example, we assume that the value of the variable (or log odds, conditional on the other variables in the imputation model) is different in those observed and unobserved by a value, δ, known as the sensitivity parameter. This is illustrated for our case study in Supplementary Figure 3. This can be achieved within MI by adding δ to the imputed values (or linear prediction of the imputed values) within each imputed data set [37].

Sensitivity analyses rely on external information about how the predictions for missing values differ from those we estimate from the observed values. This can be elicited from content experts [38] or a tipping-point analysis can be conducted, where a range of values is assumed for δ to determine how large δ would need to be to change the overall conclusion [39]. See [37,[40], [41], [42]] for more information on these approaches. The details regarding how the sensitivity analysis will be conducted and how the sensitivity parameters will be obtained should be detailed in the analysis plan.

In ALSPAC, we prespecified that the sensitivity analysis would be conducted using a pattern-mixture approach, where (after discussion with content experts) we add the fixed log odds of 0.1, 0.25, 0.5, 1, and 10 (the latter to represent an extreme MNAR mechanism) within the logistic regression model used to impute smoking status using the “offset” option within Stata's *mi impute chained* command.

### 3.2 Step 2: Conduct the preplanned analysis

#### 3.2.1 Step 2a. Examine the data

Once the data have been collected, the first step is to examine the data. This should include the following:

1. A table showing the proportion of missing data for all variables in the analysis model. Ideally this should be by variable and for the analysis as a whole. It can also be useful to explore the patterns of missing data e.g., which variables are missing together.
2. A table of the observed characteristics for the “complete” vs. “incomplete” (or all) participants or by whether variables with substantial missingness are observed.
3. An assessment of the predictors of missingness, that is, using a logistic regression model fitted to an indicator for being a complete record and predictors of missing values, that is., associations with the incomplete variables.

This examination should be used to judge the methods outlined in the analysis plan and whether the specified auxiliary variables are likely to be useful.

In ALSPAC, 3,313 of the 14,684 eligible participants (23%) had complete data on all variables required for analysis (Supplementary Table 2). Those with complete records were more likely to be first born, female, have higher educated parents, and have parents who were nonsmokers than those with incomplete data (Supplementary Table 3). After adjusting for covariates, educational attainment (the outcome) and smoking at 13 years were associated with being a complete case. This suggests that 1) a complete records analysis would have a much reduced sample size and 2) the outcome is associated with any missingness. This confirms a complete records analysis will be biased and inefficient and, because we have potentially strong auxiliary variables, MI is likely to reduce bias. It also suggests that the data may be MNAR and hence a sensitivity analysis will be important.

##### 3.2.2 Step 2b. Conduct the analysis as per the analysis plan

Once satisfied the assumptions made in the analysis plan are acceptable, the next step is to conduct the preplanned analyses. If the analysis plan needs to be revised in light of the initial data analysis, any changes should be acknowledged and justified.

In ALSPAC, data examination confirmed the methods outlined in the analysis plan are appropriate, and hence, we proceed with the preplanned MI and sensitivity analysis.

### 3.3 Step 3: Reporting

The methods section of a article should state how the missing data were addressed in the analyses (including any sensitivity analyses), including whether this was prespecified and any changes made to the prespecified plan. For each analysis, state the assumptions made and provide enough detail for the analysis to be reproducible (outlined in 1c for MI). For the sensitivity analysis, specify how this was conducted (outlined in 1d). Some of these details may appear in the supplementary material.

In the results section, the extent of missing data should be described using the summaries outlined in Step 2a, along with a summary of the reasons for the missing values if possible. Again, some of this information can be included in the supplementary material.

The inference from the various analyses should then be reported and interpreted in light of the missing data and the clinical relevance. Although the main results from sensitivity analyses should be given in the article, the full details may be presented in the supplementary material for brevity. If the results from all analyses are similar, the researcher can be reasonably confident that missing data is having little impact on the inference. In contrast, if there are contextually substantive differences, it is important to suggest an explanation for these, bearing in mind that under the MAR assumption MI should correct at least some of the biases that may arise in a complete records analyses. In this context, it should be made clear which result is likely to be the most accurate based on clinical knowledge but acknowledge the discrepancy reveals uncertainty.

Table 2 shows the results from the various analyses of our case study. These results all show strong evidence of an association between teenage smoking and lower educational attainment at 16 years, even in the extreme sensitivity analysis, when we set the sensitivity parameter to 10. Given the similarity of these results, we can be reasonably confident this is the true relationship. See the supplementary material for example text for our case study.

**Table 2:** Analysis of the relationship between smoking at 14 years and educational attainment at 16 years

Method of analysis	Number of observations in the analysis	Regression coefficient (95% CI)	*P*	% Of missing smoking values imputed as “smokers”
Primary analysis: Multiple imputation	14,684	−10.8 (−12.2, −9.4)	<0.001	13.3
Complete records analysis	3,153	−7.9 (−9.1, −6.7)	<0.001	N/A
Sensitivity analysis—sensitivity parameter = 0.1	14,684	−10.9 (−12.4, −9.4)	<0.001	14.2
Sensitivity analysis—sensitivity parameter = 0.25	14,684	−11.0 (−12.3, −9.6)	<0.001	15.5
Sensitivity Analysis – sensitivity parameter = 0.5	14,684	−11.0 (−12.3, −9.6)	<0.001	18.1
Sensitivity analysis—sensitivity parameter = 1	14,684	−10.7 (−11.8, −9.6)	<0.001	24.2
Sensitivity analysis—sensitivity parameter = 10	14,684	−4.3 (−4.7, −3.8)	<0.001	99.8

## 4 Discussion

We have proposed and illustrated a framework for the planning, analysis, and reporting of data from observational studies with incomplete data. The framework places a strong emphasis on prespecifying the analysis, including how missing data will be handled subject to a priori assumptions regarding the missingness. The full analysis plan could be published or registered for transparency. We highlight the need to assess the validity of the preplanned methods once the data are available. Finally, we encourage researchers to report the details of the analysis methodology to enable reproducibility, ideally including the statistical code, and to interpret the results based on the clinical relevance and suspected missingness mechanism. We see this framework as a useful addition to the strengthening the reporting of observational studies in epidemiology statement [43], providing additional details about dealing with missing data.

The framework encourages researchers to exploit information from auxiliary variables to recover information from incomplete observations. However, this relies on the researchers having some insight into the missingness mechanism. Therefore, when designing a study, it is important to identify plausible missingness mechanisms and plan to (i) reduce the extent of missing data during implementation as much as possible and (ii) collect data on potential auxiliary variables.

We have focused on MI to conduct MAR and MNAR analyses. One attractive feature of MI is that it separates the handling of missing data from the analysis model, so that decisions regarding the analysis model can be made without considering how the missing data will be handled. There are more elaborate ways of conducting MI, that is, using doubly robust [44] and machine learning methods [45,46] that are not considered here. In addition, MI is not always the most efficient approach and can give poor results if not carried out appropriately (i.e., using an inappropriate imputation model) [15]. There are a range of alternative methods available for conducting MAR (or MNAR) analyses, such as direct likelihood [47] and full Bayesian analysis [48]. Weighting based methods are another alternative but present their own challenges [47,49]. MI has the practical advantage of ease of (i) including auxiliary information, (ii) conducting sensitivity analyses, and (iii) handling large data sets. Irrespective of the statistical method chosen, researchers should use the steps presented here, including providing a justification for the analytical approach(es) and enough information to enable readers to repeat the analysis [43].

In some scenarios, it may be acceptable to only report results from a complete records analysis, for example, if there is strong justification for data being MCAR or covariates are the only incomplete variables, but this would need careful justification [23].

We have focused on the simple scenario of estimating an exposure-outcome relationship adjusted for confounders. The same principles would, however, apply for alternative outcomes, for example, a time-to-event outcome, alternative study designs, for example, a case-control study, or more complex analyses, for example, when dealing with multilevel data or when using propensity scores [50,51]. In all of these situations, there are additional issues that need to be considered when planning how to handle potential missing data which are beyond the scope of this manuscript. In addition, if the analysis involves particularly complex analysis models, for example, hierarchical models or splines, or specific forms of missing data, for example, in linkage data, then conducting an MAR analysis may require more sophisticated methods than presented here [28,51].

Finally, we propose using simple sensitivity analyses if required. First, this can be difficult to judge. And second, sensitivity analyses can become complex if there is missingness in multiple variables. Methods have been developed to conduct complex sensitivity analyses, for example, not at random fully conditional specification [52], and for elicitation of sensitivity parameters [53], although these are beyond the scope of this manuscript.

In summary, we have proposed an accessible framework for planning, analysis, and reporting studies with missing data. By following the framework, researchers will be encouraged to think carefully about missing data and the assumptions made during analysis and be more transparent about the potential effect on the study results. If adopted, this framework will improve the reporting standards and increase confidence in the reliability and reproducibility of published results [54].

## CRediT authorship contribution statement

**Katherine J. Lee:** Conceptualization, Methodology, Visualization, Writing - original draft. **Kate M. Tilling:** Conceptualization, Methodology, Visualization, Writing - review & editing. **Rosie P. Cornish:** Conceptualization, Methodology, Formal analysis, Visualization, Writing - review & editing. **Roderick J.A. Little:** Writing - review & editing. **Melanie L. Bell:** Writing - review & editing. **Els Goetghebeur:** Writing - review & editing. **Joseph W. Hogan:** Writing - review & editing. **James R. Carpenter:** Conceptualization, Methodology, Visualization, Writing - review & editing.
