# Supplementary material for: Framework for the treatment and reporting of missing data in observational studies: The Treatment And Reporting of Missing data in Observational Studies framework
Source: J Clin Epidemiol. 2021 Jun;134:79–88. doi: 10.1016/j.jclinepi.2021.01.008 (PMC8168830; doi:10.1016/j.jclinepi.2021.01.008)
Supplement: Supplement [file mmc1.docx]

**Supplementary material**

**Section A: Stata code to reproduce the analysis for the Avon Longitudinal Study of Parents and Children (ALSPAC) case study**

clear all

capture log close

version 15

use smoke_attain

egen miss=rowmiss(ks4pct smoke14b sex parity mumed daded dadsmoke ///

smoke ks2pct sdqtot81)

replace miss=1 if miss>1

gen complete=1-miss

save smoke_attain, replace

* Summarise characteristics of full sample and complete cases

tab1 smoke14b sex parity mumed daded dadsmoke smoke

summ ks2pct ks4pct sdqtot81, detail

tab1 smoke14b sex parity mumed daded dadsmoke smoke if complete==1

summ ks2pct ks4pct sdqtot81 if complete==1, detail

* Predictors of being a complete case

logistic complete sex

logistic complete i.parity

logistic complete i.mumed

logistic complete i.daded

logistic complete i.smoke

logistic complete dadsmoke

logistic complete sdqtot81

logistic complete ks2pct

logistic complete smoke14b

logistic complete ks4pct

*Complete case analysis

regress ks4pct smoke14b sex i.parity i.mumed i.daded dadsmoke ///

i.smoke ks2pct sdqtot81

*Multiple imputation

mi set mlong

mi register regular sex

mi register imputed ks4pct smoke14b parity mumed daded dadsmoke ///

smoke ks2pct sdqtot81 car sclasshigh housing bfduration rooms ///

eversmoke10 eversmoke13 fsmoke15 iq8 behave57

mi impute chained ///

(regress, omit((ks4pct) (ks2pct)) include((ks4pct^3) (ks2pct^2))) iq8 ///

(regress, omit((iq8)) include((iq8^(1/3)))) ks4pct ///

(regress, omit((iq8)) include((iq8^(1/2)))) ks2pct ///

(pmm, knn(5)) rooms sdqtot81 behave57 ///

(logit) bfduration housing sclasshigh car dadsmoke eversmoke10 ///

eversmoke13 smoke14b ///

(mlogit) parity daded mumed smoke fsmoke15 ///

= sex, burnin(20) add(100) rseed(5432127) augment

save smkattain_impute100, replace

mi estimate: regress ks4pct smoke14b sex i.parity i.mumed i.daded ///

dadsmoke i.smoke ks2pct sdqtot81

*Sensitivity analysis

use smoke_attain, clear

foreach delta in 0.1 0.25 0.5 1 10 {

use smoke_attain

gen msmk=mi(smoke14b)

gen sens=`delta'*msmk

mi set mlong

mi register regular sex

mi register imputed ks4pct smoke14b bfduration parity car ///

sclasshigh housing mumed daded dadsmoke smoke ks2pct eversmoke10 ///

eversmoke13 eversmoke15 fsmoke15 iq8 rooms behave57 sdqtot81

mi impute chained ///

(regress, omit((ks4pct) (ks2pct)) include((ks4pct^3) (ks2pct^2))) iq8 ///

(regress, omit((iq8)) include((iq8^(1/3)))) ks4pct ///

(regress, omit((iq8)) include((iq8^(1/2)))) ks2pct ///

(pmm, knn(5)) rooms sdqtot81 behave57 ///

(logit) sclasshigh car dadsmoke eversmoke10 eversmoke13 ///

bfduration housing ///

(logit, offset(sens)) smoke14b ///

(mlogit) parity daded mumed smoke fsmoke15 ///

= sex, burnin(20) add(10) rseed(5432127) augment

mi estimate: regress ks4pct smoke14b sex i.parity i.mumed i.daded ///

dadsmoke i.smoke ks2pct sdqtot81

save misens`delta'_imp20, replace

clear

}

**Section B: Example of the pre-specified statistical analysis plan, and the methods, results, conclusions for a paper for the ALSPAC case study**

1. **Statistical Analysis Plan**

The association between teenage smoking (at 14 years) and educational attainment at age 16 years will be assessed using a linear regression of educational attainment at age 16 years on smoking at age 14 years adjusted for the following confounders: child’s sex; parity; maternal and paternal smoking status and educational level (O level/Certificate of Secondary Education/vocational, A level^[[1]](#footnote-1)^, and degree or higher); the child’s total score on the Strength and Difficulties Questionnaire (SDQ), a measure of behavioural difficulties, measured using a parent-completed questionnaire at age 81 months; and attainment score at age 11 years (ranging from 0 to 280, converted to a percentage).

We expect there to be relatively high rates of missingness, particularly in smoking status, the exposure of interest. Missing data in ALSPAC has previously been shown to be associated with many of the covariates in the analysis model (i.e. is not MCAR). In particular, attrition and non-response have been shown to be associated with educational attainment (the outcome measure of interest in this analysis), with those with lower attainment being less likely to respond {Boyd, 2013 #506}. There are also a number of potentially useful auxiliary variables, such as smoking status at previous and later waves for imputing the smoking exposure and IQ for imputing the outcome, educational attainment. Given this, MI will be the primary method of analysis at it has the potential to reduce bias and improve precision over a complete records analysis. However, we will also conduct a complete records analysis as a comparison.

We also hypothesise that missingness in smoking at age 14 years will be associated with smoking itself, conditional on the covariates in the analysis model (i.e. MNAR), hence we will conduct a sensitivity analysis. The sensitivity analysis will be conducted using a pattern-mixture approach, where we will apply a range of sensitivity parameters within the logistic regression model used to impute smoking status. This will be incorporated using the “offset” option within Stata’s *mi impute chained* command, where (after discussion with content experts) we will add the fixed amounts of 0.1, 0.25, 0.5, 1 and 10 (the latter to represent a very extreme MNAR mechanism) to the imputed log odds of smoking to increase the log odds of smoking among those with missing smoking data.

MI will be conducted using fully conditional specification applied to all the variables in the analysis model, as well as the auxiliary variables: smoking (ever smoked) reported by the child at study clinics at age 10 and 13 years; frequency of smoking at 15 years (never, < daily and daily) reported at a study clinic; IQ measured at a study clinic at 8 years using the Wechsler Intelligence Scale for Children 3^rd^ edition (WISC-III; ref); child behaviour score at age 57 months, generated from five questions included on a parent-completed questionnaire (frequency the child bullies other children, is disobedient, tells lies, takes things belonging to others, fights with other children, each measured using a Likert-type scale from never to always); duration of breastfeeding (<3 months, 3+ months); and additional measures of socio-economic position measured during pregnancy: family occupational social class (classified as manual vs. non-manual), number of rooms in home (excluding bathrooms), housing tenure (owned/mortgaged vs rented/other) and car ownership. It is known that the attainment scores (at age 16 years, the outcome of interest, and at age 11 years, covariate) are not linearly associated with IQ. Therefore, fractional polynomials will used to obtain the best fitting non-linear model for these relationships, which will then be incorporated into the imputation model. These variables will be included as linear variables when imputing other variables. Predictive mean matching, selecting from the 5 nearest neighbours, will be used to impute both behaviour scores and number of rooms in the home because these variables are positively skewed and take on only positive values. The remaining covariates will be imputed using either logistic or multinomial logistic regression, as applicable. MI will be conducted using Stata’s mi impute chained command; 100 datasets will be imputed with a burn-in of 20 iterations.

1. **Methods, results, conclusions for a published paper**

**Methods**

We first summarised the data for the variables relevant to this analysis, including the amount of missing data, both overall and for each variable in turn.

The association between teenage smoking (at 14 years) and educational attainment at age 16 years was then assessed using a linear regression of educational attainment at age 16 years on smoking at age 14 years adjusted for the following confounders: child’s sex; parity; maternal and paternal smoking status and educational level (certificate of secondary education/vocational, O level, A level, and degree or higher); the child’s total score on the Strength and Difficulties Questionnaire (SDQ), a measure of behavioural difficulties, measured using a parent-completed questionnaire at age 81 months; and attainment score at age 11 years (ranging from 0 to 280, converted to a percentage). As per our pre-specified analysis plan, MI was used to address the missing data as the primary analysis, although as a sensitivity analysis we also present the data from a complete records analysis. Finally, we conducted a sensitivity analysis where we assumed an MNAR missingness mechanism for the exposure of interest (smoking at age 14 years). The sensitivity analysis was conducted using a pattern-mixture approach, where we applied a range of sensitivity parameters (0.1, 0.25, 0.5, 1 and 10), the latter to represent a very extreme MNAR mechanism), as an “offset” within the logistic regression model used to impute smoking status.

All MI analyses were conducted using fully conditional specification applied to all the variables in the analysis model, as well as the auxiliary variables (ever smoked at 10 and 13 years, frequency of smoking at 15 years, IQ at 8 years, child behaviour score at age 57 months, duration of breastfeeding, family occupational social class, number of rooms in home, housing tenure, and car ownership). Attainment scores (at age 16 years, the outcome of interest, and at age 11 years, covariate) were imputed using a non-linear relationship with IQ based on fractional polynomials, which resulted in attainment at age 16 years (the outcome) being imputed using linear regression dependent on the cube root of IQ and the age 11 years attainment score being imputed from the square root of IQ. Similarly, IQ was imputed from the attainment score at age 16 years cubed and attainment score at age 11 years squared. These variables were included as linear variables when imputing other variables. Predictive mean matching, selecting from the 5 nearest neighbours, was used to impute both behaviour scores and number of rooms in the home. The remaining covariates were imputed using either logistic or multinomial logistic regression, as applicable. In each case, MI was conducted using Stata’s mi impute chained command; 100 datasets were imputed with a burn-in of 20 iterations.

**Results**

Supplementary Table 2 presents a summary of the variables relevant to this research question. Importantly, only 23% of participants have complete data on all the variables required for the substantive analysis. Participants with complete records were more likely to be first born, female, to have more highly educated parents, and to have parents who were non-smokers than those with incomplete data (Supplementary Tables 2 and 3). Importantly, after adjusting for covariates, educational attainment (the outcome) (odds ratio [OR]=1.37 95% confidence interval [CI] 1.27, 1.47 per 10% increase in attainment), and smoking at 13 years (OR=0.35 95% CI 0.26, 0.47) were associated with being a complete case. This confirms a complete records analysis will be biased and, because we have auxiliary variables likely to be strongly associated with smoking and attainment, this justifies the use of MI as the primary analysis.

Table 1 in the main manuscript presents the estimated mean difference in attainment score comparing those who smoked to those who did not obtained from the primary analysis (MI), the complete records analysis and the sensitivity analyses. All of these results show that smoking at age 14 years is associated with lower educational attainment at age 16 years. This is even the case for the extreme sensitivity analysis, when we set the sensitivity parameter to 10.

**Conclusions**

There is evidence of an association between smoking at age 14 years and lower educational attainment at age 16 years.

**Section C: An example where it may be appropriate to modify the pre-specified analysis plan**

As discussed in the manuscript, it is important to follow the pre-specified analysis plan when conducting the analysis. However there are scenarios where it may be justifiable to amend the plan, namely if it becomes apparent that the assumptions made in the pre-specified plan were not correct. This could be in terms of the amount of missing data, which variables required for analysis (namely the outcome, exposure and confounders) are incomplete, or the assumed relationships between the variables in the analysis model and their missingness. One example is, if a priori it was expected that there would be very little (<5%) missing data in the exposure of interest and hence a complete records analysis was planned as the primary analysis, but when the data became available there was more missing data than expected in the exposure, and there are potentially useful auxiliary variables that are not part of the analysis model but are correlated with the incomplete exposure.

In our example, the substantial amount of missing data could mean that a complete records analysis is biased if the missingness is associated with the outcome, which might be quite likely in practice. There is also potentially useful information in the auxiliary variables which could be used to recover some of this missing data. In this context, multiple imputation is likely to provide a more reliable and efficient estimate of the exposure-outcome relationship than the pre-planned complete records analysis, and therefore would provide a superior analysis. If it was decided to amend the analysis plan in light of this post-hoc information, it would be important to acknowledge this in reporting the results from the study, and to justify the amendment to the analysis plan. The following text could be reported in the manuscript:

When planning the analysis it was expected that there would be minimal (<5%) missing data in the variables required for analysis, hence the pre-specified plan was to conduct a complete records analysis. However once the data became available it was apparent that there was more substantial missingness in the exposure of interest than expected which was associated with the outcome. There were also variables that were correlated with the incomplete which could be used to recover some of the missing information. For both of these reasons it was deemed that multiple imputation would provide a superior method of analysis and hence was adopted as the primary method of analysis.

**Section D: Supplementary tables and figures**

**Supplementary Figure 1: Sensitivity analysis where we vary the imputation distribution of the incomplete smoking exposure: (A) Relationship between the incomplete exposure and the other variables in the dataset in the observed data; (B) Relationship between the incomplete exposure and the other variables in the dataset assumed in the complete and incomplete cases under MAR, namely that the relationship is the same for all cases; (C) Relationship between the incomplete exposure and the other variables in the dataset assumed in the complete and incomplete cases in the (MNAR) sensitivity analysis where we allow these relationships to be different in those with complete and incomplete data. Note, in practice we can never know β^*. Instead we specify a sensitivity parameter, δ, or a range of δ’s, which represent the hypothesised difference between β and β^*.**

(A)

(B)

(C)

Complete and incomplete cases

Complete cases

Incomplete cases

$$\beta$$

$$\beta$$

$$\beta$$

$$\beta*$$

**Supplementary Table 1: Variables of interest in the ALSPAC case study**

| **Variable** | **Variable name** | **Values** |
| --- | --- | --- |
| Educational attainment score at 16 years (outcome) | ks4pct | 0-100% |
| Smoking at 14 years (exposure) | smoke14b | 0=non-smoker  1=current smoker |
| **Confounders** |  |  |
| Child sex | sex | 0=male; 1=female |
| Parity | parity | 0, 1, 2, 3+ |
| Maternal smoking status | smoke | 1 = never  2 = yes, but not in current pregnancy  3 = yes, including in pregnancy |
| Paternal smoking status | dadsmoke | 0 = never  1 = current or previous smoker |
| Maternal educational level | mumed | 0 = O level/CSE/vocational  1 = A level  2 = degree or higher |
| Paternal educational level | daded | As above |
| Behavioural difficulties score at 81 months | sdqtot81 | 0-40 |
| Attainment score at 11 years | ks2pct | 0-100% |
| **Auxiliary variables** |  |  |
| Smoking age 10 years | eversmoke10 | 0 = never smoked  1 = current or previous smoker |
| Smoking age 13 years | eversmoke13 | 0 = never smoked  1 = current or previous smoker |
| Frequency of smoking at 15 years | fsmoke15 | 0 = never  1 = < daily  2 = daily |
| IQ age 8 years | iq8 | 45-151 (range in data) |
| Behaviour score at 57 months | behave57 | 0-20 |
| Duration of breastfeeding | bfduration | 0 = never/<3 months  1 = 3+ months |
| Number of rooms in home (excluding bathrooms) during pregnancy | rooms | 0 to 9 |
| Family occupational social class (higher of maternal and paternal) | sclasshigh | 0 = non-manual  1 = manual |
| Car ownership | car | 0 = yes; 1 = no |
| Housing tenure | housing | 0 = mortgaged/owned  1 = private rented / other |

**Supplementary Table 2: Summary of the variables in the analysis model for the ALSPAC case study including the amount of data available for each variable, and a summary of the characteristics for the enrolled sample and those with complete records.**

| **Characteristic** |  | **Available data (n=14,684)**  **N (%)** | **Enrolled singletons and twins alive at one year and not withdrawn (n=14,684)^1^** | **Complete records**  **(n=3,313)** |
| --- | --- | --- | --- | --- |
| Sex | Male  Female | 14,684 (100%) | 7,536 (51%)  7,148 | 1,559 (47%)  1,754 |
| Parity | 0  1  2+ | 12,924 (88%) | 5,770 (45%)  4,539 (35%)  2,615 (20%) | 1,628 (49%)  1,181 (36%)  504 (15%) |
| Mother’s education | O level/lower  A level  Degree/higher | 12,412 (85%) | 8,022 (65%)  2,791 (22%)  1,599 (13%) | 1,800 (54%)  932 (28%)  581 (18%) |
| Father’s education | O level/lower  A level  Degree/higher | 10,717 (73%) | 5,445 (51%)  3,104 (29%)  2,168 (20%) | 1,473 (44%)  1,054 (32%)  786 (24%) |
| Mother’s smoking | Never smoked  Smoked, not in pregnancy  Smoking in pregnancy | 13,242 (90%) | 6,413 (48%)  3,584 (27%)  3,245 (25%) | 1,958 (59%)  934 (28%)  421 (13%) |
| Paternal smoking (ever smoked) | No  Yes | 10,690 (73%) | 4,419 (41%)  6,271 | 1,624 (49%)  1,689 |
| Behavioural difficulties score at 81 months | Median (IQR) | 7,289 (50%) | 6 (4-10) | 6 (4-9) |
| Attainment score at 11 years | Mean (SD) | 11,813 (80%) | 65% (16%) | 71% (14%) |
| Smoking at 14 years | No  Yes | 7,211 (49%) | 6,762 (94%)  449 (6%) | 3,123 (94%)  190 (6%) |
| Outcome: attainment score | Mean (SD) | 12,020 (82%) | 58% (18%) | 67% (13%) |

Note, there are 3,313 participants who have complete data on all of these variables required for analysis (23% of the original 14,684).

^1^ Denominators vary because the variables come from different sources/questionnaires and have different completion rates.

**Supplementary Table 3: Predictors of being a complete case in the ALSPAC case study (n=14,684)^1^**

| **Characteristic** |  | **Crude odds ratio (95% confidence interval)** | **Area under the curve** |
| --- | --- | --- | --- |
| Sex | Male  Female | 1.00  1.25 (1.15, 1.35) | 0.53 |
| Parity | 0  1  2+ | 1.00  0.89 (0.81, 0.98)  0.61 (0.54, 0.68) | 0.54 |
| Mother’s education | O level/lower  A level  Degree/higher | 1.00  1.73 (1.58, 1.90)  1.97 (1.76, 2.21) | 0.57 |
| Father’s education | O level/lower  A level  Degree/higher | 1.00  1.39 (1.26, 1.53)  1.53 (1.38, 1.71) | 0.55 |
| Mother’s smoking | Never smoked  Smoked, not in pregnancy  Smoking in pregnancy | 1.00  0.80 (0.73, 0.88)  0.34 (0.30, 0.38) | 0.59 |
| Paternal smoking (ever smoked) | No  Yes | 1.00  0.63 (0.58, 0.69) | 0.56 |
| Behavioural difficulties score at 81 months | For each 1 point increase | 0.96 (0.95, 0.97) | 0.55 |
| Attainment at 11 years | For each 10% increase | 1.47 (1.43, 1.51) | 0.66 |
| Smoking at 14 years | No  Yes | 1.00  0.85 (0.70, 1.04) | 0.50 |
| Outcome: attainment score | For each 10% increase | 1.67 (1.61, 1.73) | 0.70 |

^1^ Denominators in each analysis vary because vary because the variables come from different sources/questionnaires and have different completion rates.

1. CSEs (Certificate of Secondary Education) and O levels were qualifications taken at age 16 – now replaced by GCSEs (General Certificate of Secondary Education) in England, Wales and Northern Ireland. A levels are exams taken at age 18 in these countries. [↑](#footnote-ref-1)
